# Heterologous ectoine production in *Escherichia coli*: By-passing the metabolic bottle-neck

**DOI:** 10.1186/1746-1448-4-12

**Published:** 2008-08-29

**Authors:** Thorsten Bestvater, Petra Louis, Erwin A Galinski

**Affiliations:** 1 Institute of Biochemistry, Westfälische Wilhelms-Universität Münster, Wilhelm-Klemm-Strasse 2, 48149 Münster, Germany; 2 Rowett Institute of Nutrition and Health, University of Aberdeen, Greeburn Road, Bucksburn, Aberdeen AB21 9SB, UK; 3 Institute of Microbiology & Biotechnology, Meckenheimer Allee 168, Rheinische Friedrich-Wilhelms-Universität, 53115 Bonn, Germany

## Abstract

Transcription of the ectoine biosynthesis genes *ectA, ectB *and *ectC *from *Marinococcus halophilus *in recombinant *Escherichia coli *DH5α is probably initiated from three individual σ^70^/σ^A^-dependent promoter sequences, upstream of each gene. Consequently, mRNA-fragments containing the single genes and combinations of the genes *ectA *and *ectB *or *ectB and ectC*, respectively, could be detected by Northern blot analysis. Under the control of its own regulatory promoter region (*ectUp*) a seemingly osmoregulated ectoine production was observed. In addition, aspartate kinases were identified as the main limiting factor for ectoine production in recombinant *E. coli *DH5α. Co-expression of the ectoine biosynthesis genes and of the gene of the feedback-resistant aspartate kinase from *Corynebacterium glutamicum *MH20-22B (*lysC*) led to markedly increased production of ectoine in *E. coli *DH5α, resulting in cytoplasmic ectoine concentrations comparable to those reached via ectoine accumulation from the medium.

## Background

To master the osmotic stress of saline environments, halophilic organisms accumulate highly water-soluble organic osmolytes, so-called compatible solutes [1-3]. Ectoine (1,4,5,6-tetrahydro-2-methyl-4-pyrimidine carboxylic acid), the compatible solute that was first discovered in *Ectothiorhodospira halochloris *[4], is one of the most commonly found osmolytes in nature [5-9]. Besides their osmotic effect, ectoines as well as other compatible solutes have been found to improve protein folding and to protect biomolecules such as enzymes, nucleic acids, antibodies and even whole cells against heating, freeze-thawing, drying or chemical treatment [10-14]. Additional applications of ectoine include use as protective additive, modulator of proinflammatory response and moisturizer for skin care products [15-17], and potentially also for treatment of diseases related to protein misfolding [18-21]. In view of its potential as a stabilizing, protective and pharmaceutical agent, a bioprocess for ectoine production named "bacterial milking" has been developed for commercial exploitation using the halophilic eubacterium *Halomonas elongata *[22].

**Figure 1:**
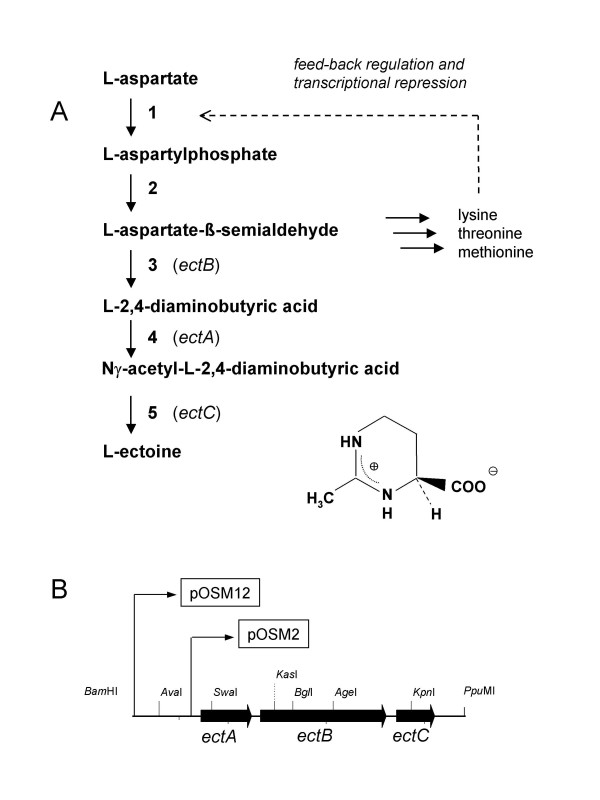
**Ectoine biosynthesis and *ectABC *gene cluster from *Marinococcus halophilus***. A: The biosynthetic pathway for ectoine [56,57] and its dependence on feed-back regulation and/or transcriptional repression of the aspartate kinases in the biosynthetic pathway of the amino acids L-lysine, L-threonine and L-methionine during heterologous expression in *E. coli*. B: Map of the ectoine biosynthetic genes from *M. halophilus *as integrated in the plasmids pOSM12 and pOSM2 (only some restriction sites are shown). In case of pOSM2 the natural promoter region upstream of *ectA *is truncated and replaced by a *lac *promoter. **1**, L-aspartate-kinase I-III; **2**, L-aspartate-β-semialdehyde dehydrogenase; **3**, L-2,4-diaminobutyric acid transaminase (*ectB*); **4**, L-2,4-diaminobutyric acid Nγ-acetyltransferase (*ectA*); **5**, L-ectoine synthase (*ectC*).

The non-halophilic *Escherichia coli *has been shown to accumulate ectoine from the surrounding medium, and as a consequence its tolerance to elevated salinities is increased [23]. Also, recombinant *E. coli *XL1-Blue is able to express the ectoine genes *ectABC *from the Gram-positive moderately halophilic *Marinococcus halophilus *and exploit the enzymes of the biosynthetic pathway for osmoregulated ectoine production [7]. The organization of the ectoine gene cluster and its relation to the ectoine biosynthetic pathway is shown in Fig. 1A and 1B. A search for consensus sequences for σ^70^/σ^A^-dependent promoters revealed two potential promoter sites upstream of *ectB*, but none at the beginning of the gene cluster [7]. Using deletion derivatives, however, the authors were able to conclude that regulating sequences must extend up to or beyond 150 bp upstream of *ectA*.

In this study, we report the transcription initiation sites of the ectoine gene cluster as determined by RACE (rapid amplification of cDNA ends) in both, the donor *Marinococcus halophilus *as well as the genetically engineered *E. coli *DH5α. In addition, we report on potential metabolic limitions for heterologous ectoine production and the generation of a new recombinant production strain freed from one such metabolic "bottle-neck" which limits substrate supply of the ectoine biosynthetic pathway.

## Results

### Northern analysis

**Figure 2:**
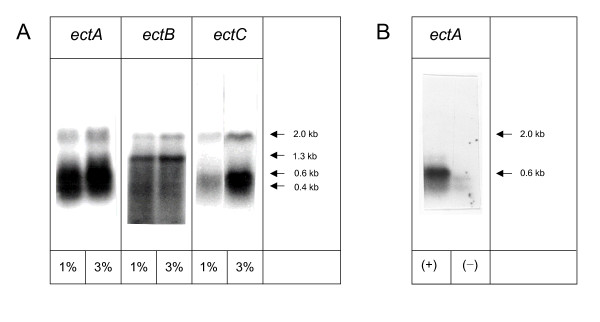
**Northern blot analysis**. Northern blot analysis of total RNA isolated from *E. coli *DH5α pOSM12 at 1% and 3% NaCl (A) and from *E. coli *DH5α pOSM2 at 3% NaCl (B) in minimal medium MM63 was performed with specific RNA probes for each of the ectoine genes *ectA*, *ectB *and *ectC *(0.4 kb = *ectC*, 0.6 kb = *ectA*, 1.3 kb = *ectB*, 1.8 kb = *ectBC*, 2.0 kb = *ectAB*). Arrows indicate where mRNA bands matching the calculated size of gene should be located. (+) and (-) refer to presence and absence of IPTG.

Expression of a genomic library of the halophilic *Marinococcus halophilus *in low-copy number vector pHSG575 [24] resulted in the identification of the gene cluster encoding ectoine biosynthetic genes *ectA*, *ectB *and *ectC *and the construction of two vectors, pOSM12 and pOSM2, enabling ectoine synthesis and enhanced salt tolerance in *E. coli *Xl-1 blue [7]. Whereas the former carries a region 720 bp upstream of the start codon of *ectA *(*ectUp*), the latter lacks regulatory elements because of a truncated upstream region (100 bp only). By Northern blot analysis with specific RNA-probes for *ectA *(approx. 0.6 kb), *ectB *(approx. 1.3 kb) and *ectC *(approx. 0.4 kb), we could identify both, mRNAs of the single gene products and mRNAs containing *ectAB *and *ectBC*, respectively, in heterologous *E. coli *DH5α pOSM12 (Fig. 2A). However we were not able to distinguish between the bands of *ectAB *(approx. 2000 bp) and *ectBC *(approx. 1800 bp) on the *ectB *blot. A band corresponding to *ectABC *mRNA with an approx. size of 2500 bp could not be detected in *E. coli *DH5α. In *E. coli *DH5α pOSM2 the transcription pattern was different with respect to *ectA*. Transcription of *ectA *or *ectAB *in DH5α pOSM2, which had its osmoregulatory DNA region upstream of *ectA *replaced by a *lac *promoter, could not be detected in the abscence of IPTG, whereas after IPTG induction, only *ectA *mRNA, but no *ectAB *mRNA was produced (Fig. 2B). Transcription of *ectB*, *ectC *and *ectBC *in DH5α pOSM2 was not influenced by IPTG and conformed with that of DH5α pOSM12.

### Determination of transcription start points by rapid amplification of cDNA ends (RACE)

**Figure 3:**
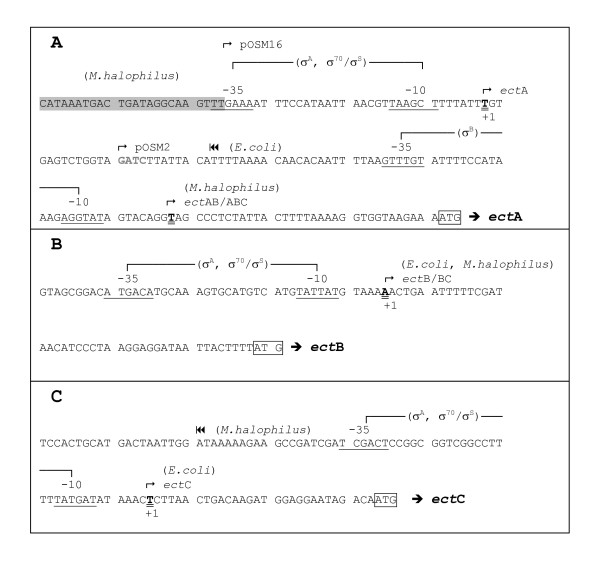
**Transcription initiation sites and putative promoter regions**. Transcription initiation sites and positions of putative σ^A^, σ^B^- and σ^70^/σ^S^-dependent promoters upstream of *ectA *(A), *ectB *(B) and *ectC *(C). The -35 and -10 regions are underlined and the start codons ATG are framed. The transcription initiation sites as determined by RACE are typed bold, underlined twice and marked (+1). The DNA sequence upstream *ectA *which is deleted in pOSM16 (see text) is underlayed grey. The *Sau*3A restriction site used for the construction of pOSM2 (↱pOSM2) is marked. |◀◀: last nucleotide of the cDNA fragment from RACE experiment, which was terminated 89 bp upstream of the start codon of *ectA *(for *E. coli*) and 83 bp upstream of *ect*C (for *M. halophilus*) (see text).

Putative transcription initiation sites for both, the donor (*Marinococcus halophilus*) and the genetically engineered acceptor (*E. coli *DH5α) were identified using the RACE technique. The identified sites are shown in Fig. 3. Subsequently potential promoter recognition sites were investigated by homology search using the Neural Network Promotor Prediction programme and assigned to the previously identified initiation sites. Primary eubacterial sigma factors are responsible for the transcription of most genes expressed in exponentially growing cells and essential for cell survival. They are known as σ^70 ^in *E. coli *and σ^A ^in *Bacillus subtilis *and other Gram-positive bacteria and have identical consensus sequences. Non-essential stationary-phase or general stress response σ-factors of the Enterobacteriaceae are called σ^S ^and similar in function (but not in consensus sequence) to σ^B ^of *B. subtilis *and related organisms [25].

In the donor (*M. halophilus*) single gene transcription of *ectA *and *ectB *appears to be under control of σ^A^-dependent promoters (Fig. 3A and 3B). The initiation sites are located 114 nucleotides and 44 nucleotides upstream of the start codon for the genes *ectA *and *ectB*, respectively. In addition to the transcription initiation sites for the single gene products, in *M. halophilus*, a site for the transcription of *ectAB *and/or *ectABC *could be found 34 bp upstream of the start codon of *ectA*. These results were obtained when the mRNA in the RACE procedure was transcribed to cDNA over two consecutive genes (approx. 0.9 kb), starting from an *ectB*-specific primer. Unfortunately, we were unable to detect an analogous transcription initiation site for *ectBC*, probably because of the length of the potential RACE product (1.6 kb for *ectBC *compared to only 0.9 kb in the case of *ectAB*). In contrast to the promoters for single gene transcription, the transcription initiation site of *ectAB *and/or *ectABC *is under the control of a putative σ^B^-dependent promoter (Fig. 3A). Taken into consideration that the primary sigma factor σ^A^ is responsible for the expression of the essential genes for cell survival, whereas σ^B ^mediates the general stress response, this could point towards an interplay of various mechanisms regulating the expression of the ectoine genes in *M. halophilus*. The RACE reverse transcription product of *ectC *mRNA appears to stop 83 nucleotides upstream of the start codon (|◀◀ in Fig. 3C), but no corresponding promoter consensus sequence could be assigned to this initiation site. Therefore, the possibility remains that this initiation site is an artefact, possibly due to the formation of a secondary structure of mRNA (terminating loop), or the result of mRNA processing. Downstream of this hypothetical transcription initiation site a potential σ^A^-dependent promoter site, comprising a -10 and a -35 region, could be found (Fig. 3C) with a potential transcription initiation point 29 bp upstream of *ectC*. This promoter site is apparently not used in *M. halophilus *under the conditions employed, it is however recognized by the genetically engineered host, *E. coli *DH5α.

In *E. coli*, transcription of each single gene *ectA*, *ectB *and *ectC *is under control of a σ^70^/σ^A^-dependent promoter with both -10 and -35 region. Transcription is initiated 29 bp upstream of the start codon for *ectC*, 44 bp for *ectB *and probably 114 bp for *ectA *(Fig 3). In the case of *ectA*, experimental proof of the predicted transcription initiation site posed a problem as resulting cDNA fragments were terminated 89 bp upstream of the start codon (|◀◀ in Fig. 3A). No suitable promoter sequence was found in this region. Even though the experimental characterization of a transcription initiation site upstream of *ectA *was not successful, we were able to reveal the likely initiation site shown in Fig. 3A from the data available. Louis and Galinski [7] were able to demonstrate that a deletion 146 bp upstream of the start codon (shaded area in Fig. 3) resulted in unregulated ectoine production in *E. coli *XL1-Blue pOSM16. In this work we demonstrated, using DH5α pOSM2, that a deletion 100 bp upstream of the start codon (↱ pOSM2 in Fig. 3A) completely disabled transcription of *ectA*. From this we concluded a promoter region beyond 100 bp and somewhere around 150 bp upstream of *ectA*, probably identical to the σ^A^-dependent site identified for *M. halophilus*. (Fig. 3A). This proposal is corroborated by the finding that the deletion in pOSM16 changes the first two thymidine nucleotides (TT) of the sequence of the potential σ^70^-dependent -35 region TTGAAA (Fig. 3A). The consensus sequence for this -35 region is TTGACA [25].

### Growth and ectoine production of genetically engineered *E. coli*

**Figure 4:**
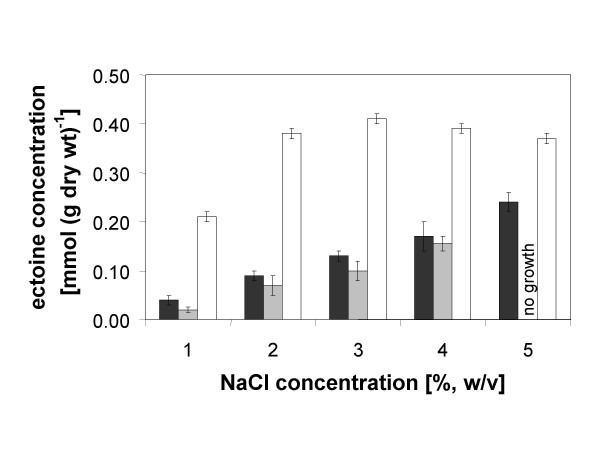
**Intracellular ectoine content (heterologous production vs. uptake)**. Intracellular ectoine concentrations of the recombinant ectoine producers *E. coli *DH5α pOSM12 (black bars) and pOSM2 (grey bars), the latter supplemented with IPTG for induction of the *lac *promoter upstream of *ectA*, and of the control strain *E. coli *DH5α pHSG575, supplemented with 2 mM ectoine in the growth medium, (white bars) at salinities between 1% and 5% NaCl in minimal medium MM63. Mean values and standard deviations are based on three independent experiments.

Plasmid pOSM12 carries a 3.5 kb DNA fragment from *M. halophilus *containing the ectoine biosynthetic genes *ectABC *and a putative promoter region (*ectUp*) 720 bp upstream of *ectA*, whereas on pOSM2 this upstream region is deleted 100 bp short of *ectA *and substituted by a *lac *promoter (Fig. 1B). Expression of the ectoine biosynthetic genes *ectABC *in *E. coli *DH5α using both pOSM12 and pOSM2, the latter supplemented with IPTG, led to an accumulation of ectoine in the cells (Fig. 4). The amount of intracellular ectoine increased with salinity of the growth medium in both cases but appeared to be slightly lower in the strain DH5α pOSM2. The most obvious difference was that the *E. coli *construct devoid of the promoter region (pOSM2) was unable to grow at 5% NaCl (Fig. 4). The levels of ectoine accumulation were, however, always lower than those of the control strain (DH5α pHSG575) in the presence of externally supplied ectoine (Fig. 4).

**Figure 5:**
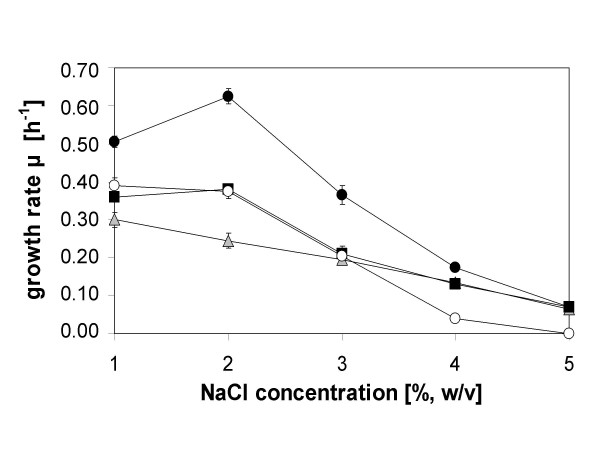
**Maximum growth rates**. Maximum growth rates [h^-1^] of the recombinant ectoine producer *E. coli *DH5α pOSM12 (▲) and of the control strain *E. coli *DH5α pHSG575, with (●) and without (○) supplementation of 2 mM ectoine at salinities of between 1% and 5% NaCl in minimal medium MM63. The novel construct pAKECT1 (■) employing deregulated aspartate kinase from *C. glutamicum *(induced with 0.5 mM IPTG) displayed improved growth at salinities above 3% NaCl. Mean values and standard deviations are based on three independent experiments.

Growth rates of DH5α pOSM2 were similar to those of the unsupplemented control strain DH5α pHSG575 without the ectoine biosynthetic genes (data not shown), whereas DH5α pOSM12 displayed slower growth than the control at salinities below 3% NaCl (Fig. 5). At salinities above 3% NaCl, however, a growth promoting effect was observed, which enabled the organism to tolerate up to 5% NaCl (Fig. 5). Although the intracellular concentration of ectoine increased linearly with salinity, it remained below ectoine levels achieved by the control strain DH5α pHSG575 through accumulation from the medium (Fig. 4). In the latter, ectoine accumulation increased up to a salinity of 3% to a final value of approx. 0.4 mmol (g dry weight)^-1^, which then remained constant at salinities between 3 and 5% NaCl, resulting in a strong promotion of cell growth (Fig. 5). Comparison with *Halomonas elongata*, an extremely halotolerant ectoine-producing bacterium, revealed that the intracellular ectoine levels were similar up to 3% NaCl but approximately 1.5-fold higher in *H. elongata *at 5% NaCl (data not shown). This indicates that *E. coli *DH5α, when grown in an ectoine-containing medium, is able to establish the required ectoine levels in a salinity range of 1–3% NaCl through uptake mechanism, but not at higher salinities. The heterologous ectoine production, on the other hand, appears to be restricted, leading to markedly lower ectoine levels in the cells (Fig. 4).

The comparatively low ectoine levels in recombinant *E. coli *DH5α pOSM12 could not be explained by limitation of the cells' capacity because the ectoine-accumulating control strain tolerated higher intracellular ectoine concentrations nor by leakage because ectoine was not detectable in the medium (HPLC sensitivity limit: 10 μM). In addition, higher cytoplasmatic ectoine levels caused a significant growth-promotion of the control strain and appeared to have no negative effect on the cells. We therefore assumed that regulatory mechanisms in the metabolism of *E. coli *caused the limitation of ectoine production in the recombinant strains. To determine potential candidates for the limiting steps, the ectoine biosynthetic pathway had to be analyzed in context with *E. coli's *metabolic network, where L-aspartate β-semialdehyde, the substrate of the first enzyme of the ectoine biosynthetic pathway, is an intermediate of the biosynthetic pathway of the amino acids of the aspartate family (Fig. 1A). As *E. coli *has three aspartate kinase activities (I-III), which are regulated by feedback inhibition and/or transcriptional repression [26], we suspected a bottle-neck for the supply of this metabolic precursor in *E. coli *DH5α pOSM12.

### Role of aspartate kinases in ectoine synthesis

**Figure 6:**
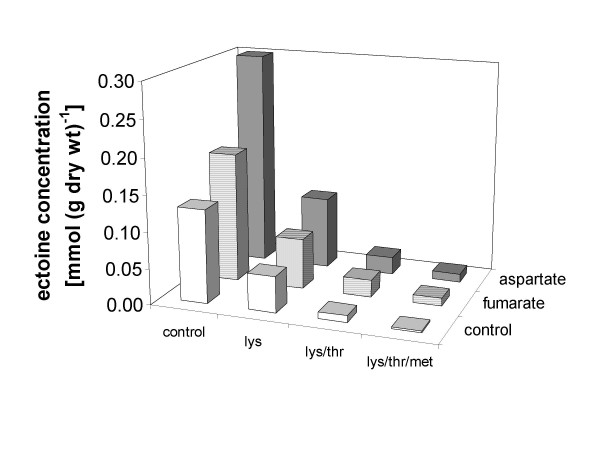
**Influence of feed-back inhibitors and precursors**. Intracellular ectoine concentrations of *E. coli *DH5α pOSM12 in minimal medium MM63 at 3% NaCl, as influenced by supplementation with the feedback-inhibitors and transcriptional repressors L-lysine (lys), L-threonine (thr) and L-methionine (met) (each 1 mM), or with the substrate L-aspartate (1 mM) and its precursor fumarate (1 mM). Control experiments were performed in the absence of regulating amino acids.

In order to obtain more information about the presumed bottle-neck in *E. coli *DH5α pOSM12, growth experiments at 3% NaCl were performed in the presence of the end products and regulators of the synthesis of the aspartate family amino acids: L-lysine, L-threonine and L-methionine (each 1 mM). In the presence of these amino acids, ectoine biosynthesis was significantly reduced, leading to an almost complete inhibition by a mixture of all three amino acids (Fig. 6). In contrast, the addition of L-aspartate (1 mM), the substrate of the aspartate kinases, or its precursor fumarate (1 mM) to the growth medium resulted in 2.3-fold and 1.4-fold elevated ectoine levels, respectively. Higher L-aspartate or fumarate concentrations in the medium did not cause a further increase in ectoine production. As expected, the enhancing effect of aspartate or fumarate on ectoine biosynthesis was again drastically reduced in the presence of L-lysine, L-threonine and L-methionine (Fig. 6).

### Opening the bottle-neck with plasmid pAKECT1

The results obtained from the supplementation experiments provide strong evidence that the aspartate kinases, which are the key regulatory enzymes for the biosynthetic pathway of aspartate family amino acids in *E. coli*, represent a bottle-neck for ectoine production in the non-halophilic host DH5α pOSM12 because of stringent feed-back inhibition and/or transcriptional repression. Our strategy to relieve this metabolic restriction was to alter the regulation of the biosynthetic pathway by co-expression of the ectoine biosynthetic genes together with the feedback-insensitive aspartate kinase (*lysC*) from *C. glutamicum *MH20-22B, which had already been successfully expressed in *E. coli *[27,28].

**Figure 7:**
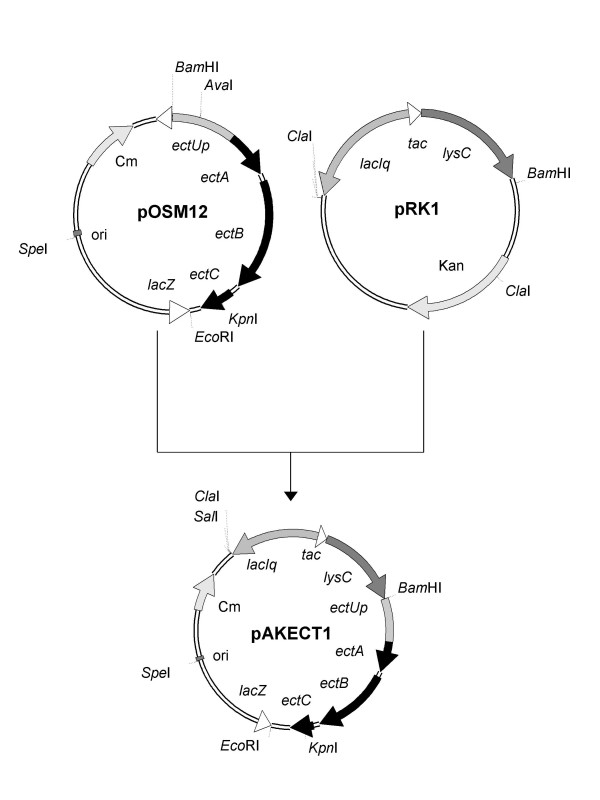
**Construction of the plasmids pAKECT1**. Plasmid pAKECT1 (10.1 kb) was constructed from pOSM12 and pRK1. Only donor plasmids, final construct and the relevant restriction sites are shown. Due to lack of suitable restriction sites, a complex construction scheme had to be applied (details in text). *ectUp*: region upstream of *ect*A with putative osmoregulated promoter sequences. *lysC*: deregulated aspartate kinase from *Corynebacterium glutamicum *MH20-22B.

**Figure 8:**
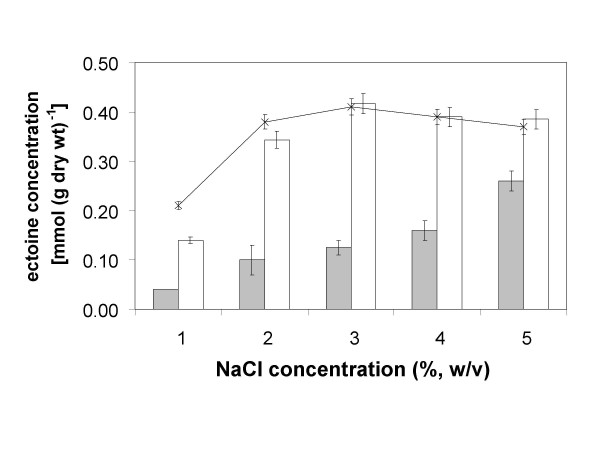
**Improved intracellular ectoine content in *E. coli *DH5α pAKECT1**. Intracellular ectoine concentrations of the new recombinant ectoine producer *E. coli *DH5α pAKECT1 with (white bars) and without (grey bars) IPTG-induction of the feedback-insensitive aspartate kinase at salinities between 1% and 5% NaCl in minimal medium MM63. For sake of comparison the data obtained with the control strain *E. coli *DH5α pHSG575, supplemented with 2 mM ectoine in the growth medium, are added to the graph as a solid line. Mean values and standard deviations are based on three independent experiments.

The plasmid pAKECT1 (Fig. 7) contains *lysC *under the control of a *tac *promoter and the gene cluster *ectABC *with the putative osmoregulated promoter sequence upstream of *ectA *(*ectUp*), just as in pOSM12. The possibility to separately induce the ectoine biosynthetic genes by osmotic stress and the aspartate kinase by IPTG enabled us to directly investigate the effect of the deregulated aspartate kinase on growth rates and ectoine production. As shown in Fig. 5, induction of aspartate kinase increased the growth rate at low salinity (1% and 2% NaCl) to the same level as the control. Without IPTG-induction of the feedback-insensitive aspartate kinase gene the ectoine levels in the cells were similar to those in DH5α pOSM12, but upon addition of IPTG, ectoine production increased approx. 3-fold in the range of 1–3% NaCl and reached a maximum of 0.4 mmol (g dry weight)^-1^, which remained relatively constant at salinities of 3% NaCl, to 5% NaCl (Fig. 8). The observed saturation level at 3% NaCl and higher perfectly correlated with the levels achieved by ectoine uptake from the growth medium (Fig. 4). This observation provides strong evidence that we succeeded in by-passing the regulatory mechanisms which caused the metabolic restriction for ectoine production in recombinant *E. coli *DH5α.

### Enzymatic activity of the aspartate kinases

The enzyme assay for aspartate kinases proved the presence of a feedback-insensitive aspartate kinase from *C. glutamicum *MH20-22B (Table 1). In *E. coli *DH5α pAKECT1 the enzyme activity after IPTG induction was about 2-fold higher than in pOSM12 and pOSM2. Furthermore, no inhibition by L-lysine and L-threonine occurred in the strains expressing deregulated aspartate kinase, whereas *E. coli *aspartate kinases from DH5α pOSM12 and DH5α pOSM2 displayed a 55% lower activity after addition of L-lysine and L-threonine (10 mM in the assay mixture). The fact that aspartate kinase activity in *E. coli *DH5α pAKECT1 is the same, both in the presence and absence of the inhibitors (L-lysine and L-threonine) indicates that a deregulated aspartate kinase from *C. glutamicum *leads to increased levels of aspartate family amino acids, and that as a consequence, the contribution of *E. coli *aspartate kinases I, II and III is insignificant, probably due to complete inhibition/repression under these conditions.

**Table 1:** Aspartate kinase activity

Strain	specific activity [nmol (min mg protein)^-1^]	
	
	No addition	Lys/Thr
*E. coli *DH5α pOSM12	5.4 ± 0.2	2.4 ± 0.1
*E. coli *DH5α pOSM2	5.2 ± 0.2	2.2 ± 0.1
*E. coli *DH5α pAKECT1	10.0 ± 0.4	10.1 ± 0.5

Specific aspartate kinase activity in recombinant ectoine producing *E. coli *pAKECT1 and its inhibition by L-lysine and L-threonine at a final concentration of 10 mM in the assay mixture. Mean values and standard deviations are based on three independent experiments.

## Discussion

### Transcription of ectoine genes in *M. halophilus *and recombinant *E. coli *DH5α

Data on transcription regulation are still rare for halophilic eubacteria, but have been investigated in detail for the Gram-positive non-halophilic model organism *Bacillus subtilis*, and, of course, *E. coli*. Transcription of genes essential for cell survival during exponential growth is controlled by the primary sigma factors σ^A ^and σ^70^, respectively, which recognize a promoter consensus sequence comprising a -35 region TTGACA, a spacer of 16–18 nucleotides and a -10 region TATAAT [25]. Of the three σ^A^/σ^70 ^promoters identified in this study, only the one upstream of *ectB *(ATGACA-N_18_-TATTAT) had previously been identified by Louis & Galinski [7], because the other two (TTGAAA-N_17_-TAAGCT and TCGACT-N_17_-TATGAT) deviated by 4 and 3 nucleotides, respectively, from the consensus sequence.

General stress response, on the other hand, is mediated by the alternative sigma factor σ^B ^in *Bacillus subtilis *(among other Gram-positive bacteria) and σ^S ^in *E. coli *[29,30]. For σ^B^-dependent promoters a -35 region GTTTAA and a -10 region GGGTAT, separated by a spacer of 12–14 nucleotides, have been proposed [31]. For σ^S^-dependent promoters in *E. coli *Espinosa-Urgel et al. [32] proposed a -10 region CTATACT, which is only slightly different from the -10 region recognized by σ^70^. A conserved -35 region could not be defined so far, but an intrinsic curvature in this region is proposed to compensate for it. Due to these marked differences it is rather unlikely that a genuine σ^B^-dependent promoter of a Gram-positive bacterium should be recognized by the Gram-negative *E. coli*.

In earlier work Louis and Galinski [7] could not resolve the question whether the three ectoine biosynthetic genes (*ectA*, *ectB*, *ectC*) are transcribed separately or as a single operon. Using the RACE method we have now shown that they are transcribed both as single genes and as mRNA's comprising *ectAB*, *ectBC *and possibly *ectABC *in *M. halophilus *(not shown). Heterologous expression of the ectoine gene cluster in *E. coli*, also resulted in single and double gene mRNA products.

We have shown here the successful expression of the ectoine biosynthesis genes *ectA, ectB *and *ectC *(as well as *ectAB *and *ectBC*) from the Gram-positive *M. halophilus *in the Gram-negative *E. coli*. This is explained by recognition of all three σ^A^-dependent promoters preceeding individual genes of the ectoine biosynthesis gene cluster of *M. halophilus*. Due to the conformity of the consensus sequence of σ^A^- and σ^70^-dependent promoters this result is not surprising. In addition to the σ^A^-dependent promoters, a σ^B^-dependent promoter for the transcription of *ectAB *(and possibly *ectABC*) could be characterized upstream of *ectA*, suggesting that transcription of the single and the multiple gene products is initiated via different regulatory mechanisms in the donor *M. halophilus*. This promoter recognition sequence (GTTTGT-N_13_-AGGTAT) deviates by 3 nucleotides from the consensus sequence and had, therefore, previously not been recognized by Louis and Galinski [7]. A potential σ^B^-dependent promoter 280 bp upstream of *ect*A, which was proposed by Louis and Galinski [7], is apparently not involved in the regulation of transcription of ectoine genes under the experimental conditions employed. Use of the σ^B^-dependent promoter by recombinant *E. coli *was neither demonstrated nor to be expected. Due to the similarity of the σ^S^-dependent -10 region CTATACT to a σ^70^-dependent promoter, it cannot be stated without further investigation, e.g. by sigma-factor binding studies, whether transcription of ectoine biosynthetic genes in *E. coli *is under control of σ^70^, σ^S ^or an interplay of both. In addition it has already been shown in several studies that σ^70^-dependent promoters could also be recognized by σ^S ^[33,34], and a regulatory interplay of σ^70 ^and σ^S^, based on changes in binding affinity affected by global regulatory factors, was proposed [35,36].

Recent work on *Bacillus pasteurii *has shown that the *ectABC *genes are organised in a single operon in this organism. Expression of ectoine genes was only observed when cells were grown at elevated osmolarity and a single gene transcript (2.6 kb) and a typical σ^A^-dependent promotor region were identified [37]. Unfortunately upshock experiments were not conducted, hence the possibility still remains that an additional stress response promoter (σ^B^-dependent) may also be involved in the organism's short-term adaptation. In contrast to *B. pasteurii *(a halotolerant species) *M. halophilus *is a true halophile with a growth optimum at around 2 M salt. It is therefore not surprising that its salt stress response strategies are apparently more complex.

In a comprehensive promoter analysis of the ectoine gene cluster in *Chromohalobacter salexigens *(member of the *Halomonadaceae*) four putative transcription initiation sites were identified, at 44, 96, 134 and 149 bp upstream of the *ect*A start codon. Two of these were of the σ^70^-type, one probably σ^S ^and a fourth promoter with no similarity to known sigma factors. Consequently, the authors concluded the existence of a complex regulation pattern of ectoine synthesis in this true halophile [38].

The observation of osmoregulated ectoine production in *E. coli *DH5α pOSM12 could of course result from posttranscriptional regulation, including for example controlled uptake and/or excretion. However, as both transport systems for ectoine (ProP and ProU) are functional in *E. coli *DH5α pOSM12 and ectoine was not detected in the medium (at 10 μM sensitivity), we propose that ectoine synthesis is, at least partly, controlled at the level of enzyme activity. Still, the inability of *E. coli *DH5α pOSM2 to transcribe *ectA *and *ectAB *in the absence of IPTG and *ectAB *even under IPTG-induction, as well its impaired ectoine production, stresses the importance of the DNA region upstream of *ectA *(*ectUp*) for controlled expression of the ectoine biosynthesis genes. A transcriptional fusion of this promoter region (*ectUp*) with the reporter gene *gfp *was shown to be osmotically induced in *E. coli *and, more importantly, down-regulated in the presence of externally supplied compatible solutes [39]. The promoter region upstream of *ectA*, therefore, appears to sense a regulatory signal, apparently common for both the Gram-positive *M. halophilus *and the Gram-negative *E. coli*.

### Opening up of a metabolic bottle-neck for ectoine production

Successful heterologous expression of the ectoine biosynthetic genes from the halophilic *M. halophilus *in *E. coli *XL1-Blue by Louis and Galinski [7] enabled us for the first time to utilize genetically engineered strains for ectoine production. However, as *E. coli *XL1-Blue in medium MM63 displayed a narrow salinity range (1–3% NaCl) and growth rates were by a factor of 2–5 lower than with DH5α, experiments in this study were conducted with *E. coli *DH5α. The pivotal role of *E. coli *aspartate kinases as a limiting metabolic bottle-neck had been realised before, as shown by the use of *E. coli *feedback-insensitive aspartate kinase to enhance threonine production in transgenic alfalfa (*Medicago sativa *L.) [40]. The data presented here prove that stringent feedback-regulation and/or transcriptional repression of the aspartate kinases in *E. coli *is in fact also the main limiting factor for recombinant ectoine production in this host.

When growth rates of *E. coli *DH5α pAKECT1 are compared to those of the control which accumulates ectoine from the medium (Fig. 5), one can see that the ectoine-synthesizing construct has a 40% lower growth rate at 2% and 3% NaCl. This difference may be explained by energy requirements and side-effects of a deregulated amino acid metabolism within the aspartate family. However, as this difference is diminished at higher salinities and completely abolished at 5% NaCl, one may conclude that, at the upper range of salt tolerance, ectoine production in the genetically engineered strain is equally efficient as ectoine uptake and that growth-limitations caused by overexpression of foreign genes and overproduction of aspartate family amino acids become less important.

As highest cytoplasmic ectoine concentrations (0.4 mmol (g dry weight)^-1^) were already achieved at a medium salinity of only 3% NaCl, this could be seen as an opportunity for ectoine production at comparatively low salt concentrations and relatively high growth rate (μ = 0.21, t_d _= 3.3 h) [41]. Louis and Galinski [7] have previously reported similar cytoplasmic ectoine levels (0.38 mmol (g dry weight)^-1^) in recombinant *E. coli *XL1-Blue (containing plasmid pOSM11) at 3% NaCl. This strain however has a much lower growth rate (approx. 0.1) under the same conditions and appears to experience other growth-limiting restrictions. In order to improve the space-time yield of heterologous ectoine production even further, future work will address the option to combine the ectoine biosynthetic gene cluster with its corresponding genuine aspartate kinase from *M. halophilus*. The chances are that this enzyme will be feed-back regulated and/or transcriptionally repressed when osmotic equilibrium is achieved. Unfortunately, this postulated gene has so far not been identified in *M. halophilus*.

Co-expression of ectoine biosynthetic genes from *M. halophilus *and feedback-insensitive aspartate kinase from *C. glutamicum *MH20-22B in *E. coli *DH5α pAKECT1 resulted in strongly elevated ectoine levels, which correlated with the levels obtained when ectoine was accumulated from the growth medium (0.4 mmol (g dry weight)^-1^). A most important observation during all our studies with *E. coli *DH5α pAKECT1 was that ectoine levels increased only up to 3% NaCl (as in accumulating cells) and that ectoine was not detected in the growth medium at the end of the experiments. In case of unregulated synthesis an efflux of the overproduced ectoine via mechanosensitive channels (Msc) would have been conceivable [42-44]. The above conclusion appears to stand in contrast to the findings by Schubert et al. [45] who demonstrated continuous excretion of ectoine from a transgenic *E. coli*. The authors introduced the ectoine gene cluster from *Chromohalobacter salexigens *(devoid of the promoter region) into *E. coli *DH5α under the control of a *tet *promoter. Following a high-cell density fermentation to 20 g L^-1 ^(cell dry weight) and subsequent induction, they observed continuous excretion of ectoine at a rate of 2 mg g^-1 ^h^-1^, while the cellular level of ectoine stayed low (5 mg (g dry weight)^-1^). Such a low leakage rate would not have been detected under the low-cell density conditions employed here. Another experimental difference of the work reported here, besides the different origin of the genes, is transcriptional control by the orginal promoter region (*ectUp*) and use of growth conditions, under which compatible solute uptake systems are activated.

It is intriguing that ectoine levels were nearly identical in accumulating cells with ectoine in the growth medium, and in synthesizing cells harbouring pAKECT1. The only viable conclusion seems to be that heterologously expressed ectoine biosynthetic enzymes of *M. halophilus *are, according to osmotic needs, tuned and regulated in the phylogenetically distant host *E. coli*. This phenomenon may be explained by allosteric regulation of gene products at the level of enzyme activity, caused by yet unkown general osmotic response mechanisms shared by a large range of different bacteria.

## Conclusion

In conclusion, we demonstrated that a metabolic bottle-neck for ectoine production in the non-halophilic recombinant *E. coli *DH5α can be relieved by coexpression of a deregulated aspartate kinase from *C. glutamicum*, and in doing so we paved the way for alternative, economically viable production methods. The surprising observation, however, that heterologous expression of the ectoine biosynthetic genes does not lead to overproduction in the host under the conditions employed, stresses the need to investigate regulatory mechanisms at enzyme level in order to disclose the biochemical signal which indicates osmotic balance to the cell.

## Methods

### Organisms, growth conditions and plasmids

*Marinococcus halophilus *DSM 20408^T ^was grown at 37°C in complex medium FP5 or FP10 consisting of 1.47% (w/v) liquid fish peptone S490 (Primex AS, Norway), 10 g L^-1 ^glucose · H_2_O, 2 g L^-1 ^NH_4_Cl, 0.5 g L^-1 ^K_2_HPO_4 _and either 45 g L^-1 ^NaCl and 5 g L^-1 ^artificial sea salt (FP5) or 90 g L^-1 ^NaCl and 10 g L^-1 ^artificial sea salt (FP10). Glucose and K_2_HPO_4 _were autoclaved separately and added to the medium after cooling.

*E. coli *DH5α (F^- ^ø80d*lac*ZΔM15 Δ(*lac*ZYA-*argF*)U169 *endA1 recA1 hsdR17*(r_K_^- ^m_K_^+^) *deoR thi-1 supE44 *λ-*gyrA96 relA1*) and XL1-Blue (*recA1 endA1 gyrA96 thi-1 hsdR17 supE44 relA1 lac*(F^+ ^*proAB lacI*^q ^ZΔM15 Tn*10*) were grown aerobically at 37°C either in Antibiotic Medium No.3 (Oxoid, Wesel, Germany) or in minimal medium MM63 [46] with 3.0 ml L^-1 ^vitamin solution [47] and 1–5% NaCl. For selection of cells harbouring the plasmid pHSG575 [24] or derivatives, chloramphenicol was added to the medium at a final concentration of 25 μg ml^-1^. For supplementation experiments, the medium MM63 with 3% NaCl contained 1 mM L-lysine, L-threonine, L-methionine, L-aspartic acid or fumaric acid. For induction of the *lac *and *tac *promoter on plasmid pOSM2 and pAKECT1, respectively, the medium contained 1 mM IPTG. The cells were harvested by centrifugation (5000 g; 4°C) and freeze-dried.

Plasmid pRK1 containing the gene *lysC *from *Corynebacterium glutamicum *MH20-22B was kindly provided by Lothar Eggeling (FZ Jülich, Germany). Plasmids pOSM2 and pOSM12, comprising vector pHSG575 and DNA fragments encoding the ectoine gene cluster, were isolated from *E. coli *XL1-Blue pOSM2 and pOSM12 [7,24,48].

### Northern analysis

Total RNA was isolated from exponentially growing cells using the High Pure RNA Isolation Kit (Boehringer, Mannheim, Germany) according to the recommendation of the manufacturer. Northern blots were performed following standard methods [49], except for using DIG-labeled RNA probes, produced with the DIG RNA Labeling Kit (Boehringer, Mannheim, Germany), at 68°C for prehybridisation and hybridisation. After blocking the membrane and binding of anti-DIG-alkaline phosphatase conjugate (Boehringer, Mannheim, Germany) to the DIG-labeled RNA, chemoluminescence of CDP-Star™ (Boehringer, Mannheim, Germany) was detected by exposure of the membrane to a chemiluminescence film.

### Rapid amplification of cDNA ends (RACE)

Identification of putative transcription initiation sites was performed by RACE, according to the method of Bertoli and Burrows [50], using 5 μg isolated total RNA and the first strand reverse transcription primers (RT primers) and PCR primers shown in Table 2. The amplified cDNA ends were cloned into the plasmid pGEM^®^-T (Promega, Mannheim, Germany) and sequenced by GATC (Koblenz, Germany). Each of the identified transcription initiation sites was confirmed in three independent experiments.

**Table 2:** Primers used for the rapid amplification of cDNA ends (RACE)

Primer	Sequence	Target
*Race A1*	5'-GGAATGAAGGCCGTTACGAA-3'	*ectA *(RT primer)
*Race A2*	5'-ACGATTGAATCGACGGAACC-3'	*ectA *(PCR primer)
*Race B1*	5'-TGCCGTGGAAGCCATTAGTA-3'	*ectB *(RT primer)
*Race B2*	5'-CCGTCTTCCTGAATATAGGT-3'	*ectB *(PCR primer)
*Race C1*	5'-ACGCCTGGTTCCAGCTGATA-3'	*ectC *(RT primer)
*Race C2*	5'-CCGGCACGAATAATTGTGTC-3'	*ectC *(PCR primer)
*dG_15_*	5'-TAGATCTAGAGCTCGGGGGGGGGGGGGGG-3'	oligo-dC tail

### DNA manipulation

Plasmid isolation from *E. coli *was performed using the GFX™ Micro Plasmid Prep Kit (Amersham, Braunschweig, Germany). DNA fragments were isolated from agarose gels with the Silica Spin Fragment DNA Kit (Biometra, Göttingen, Germany). Restriction digests, ligations and PCR reactions were performed according to the recommendations of the enzyme manufacturer (New England Biolabs, Schwalbach, Germany). Transformation of *E. coli *was carried out by the calcium chloride/rubidium chloride method [51,52]. Sequencing was performed by GATC (Konstanz, Germany).

### Construction of the plasmid pAKECT1

The construction of plasmid pAKECT1 (Fig. 7) was performed in three steps. First the *ectC *DNA sequence was amplified from the plasmid pOSM12 using PCR primers, which created an additional *Xma*I restriction site upstream and additional *Bam*HI, *Cla*I and *Sal*I restriction sites downstream of *ectC*. After subcloning into the vector pGEM-T^® ^(Promega, Mannheim, Germany) and sequencing, *Xma*I- and *Sal*I-digested *ectC *was ligated to *Xma*I- and *Sal*I-digested pHSG575 [24]. In a second step the DNA fragment of plasmid pRK1 (Fig. 7), containing *lysC *under the control of a *tac *promoter and *lacI*^*q*^, was ligated to this plasmid downstream of *ectC*, using *Cla*I and *Bam*HI restriction sites. From the resulting intermediate plasmid a DNA fragment, containing the genes *lysC *and *lacI*^*q*^, was re-cut by *Bam*HI- and *Spe*I-digest and ligated to *Bam*HI- and *Spe*I-digested pOSM12 (Fig. 7).

The ectoine gene cluster in pAKECT1 is under the control of its own regulatory promoter region (*ectUp*), whereas *lysC *is under the control of a *tac *promoter. Consequently, we were able to separately induce ectoine synthesis via osmotic stress and deregulated aspartate kinase activity via isopropyl-β-D-thiogalactopyranoside (IPTG).

### Analytical Methods

Approx. 30 mg of freeze-dried cell material from an exponentially growing shaking culture (medium MM63) was used for the extraction of intracellular solutes according to a modification of the method of Bligh and Dyer [53] with methanol/chloroform/water (10:5:4) as described previously [54]. The cell extracts were analysed by isocratic HPLC using a GromSil^® ^aminopropyl column (Grom, Herrenberg, Germany) and acetonitrile/water (75:25 v/v) at a flow rate of 1 ml min^-1 ^as the mobile phase. For^ 13^C-NMR analysis of cell extracts 1.5 g freeze-dried cell material was processed as above. The polar phase was evaporated to dryness at 70°C and dissolved in 1 ml D_2_O supplemented with 10 mg 3-(trimethylsilyl) propionic acid sodium salt (TMSP) as an internal reference and 30 μl acetonitrile as internal standard.^ 1^H -decoupled ^13^C-NMR spectra relative to TMSP were recorded in pulsed Fourier-transform (FT) mode on a Bruker ARX 400 spectrometer operating at 100.62 MHz (^13^C) and 400 MHz for the proton channel.

### Enzyme assays

Aspartate kinase was assayed in extracts of cells resuspended in 50 mM (NH_4_)_2_SO_4_, 50 mM triethanolamine, 5 mM dithioerythritol, 1 mM EDTA pH 7.5 according to the method described by Black and Wright [55]. The assay mixture was composed of 100 mM Tris/HCl pH 7.5, 14.5 mM ATP, 42 mM MgCl_2_, 431 mM (NH_4_)_2_SO_4_, 613 mM NH_2_OH·HCl, 95 mM sodium L-aspartate and 125 μl of extract in a total volume of 1.2 ml. After incubation at 30°C for 30 minutes the reaction was stopped by the addition of 600 μl 3.8% (w/v) FeCl_3_. 6H_2_O and 5.8% trichloroacetic acid in 1.4 M HCl. After centrifugation the absorbance of the assay mixture at 546 nm was measured and compared to a calibration curve obtained with aspartyl hydroxamate. Total protein concentrations were determined using the BCA (bicinchoninic acid) Protein Assay Kit (Pierce, Rockford, USA) according to the recommendations of the manufacturer.

## Competing interests

The authors declare that they have no competing interests.

## Authors' contributions

TB carried out the molecular, physiological and analytical work and drafted the manuscript. PL created plasmid pOSM12, initiated the work on heterologous expression of ectoine biosynthetic genes in *E. coli *and contributed to the work on the metabolic bottle-neck. EAG conceived of the study, coordinated the work and revised the manuscript. All authors read and approved the final version of the manuscript.
